# Fermented-Food Metagenomics Reveals Substrate-Associated Differences in Taxonomy and Health-Associated and Antibiotic Resistance Determinants

**DOI:** 10.1128/mSystems.00522-20

**Published:** 2020-11-10

**Authors:** John Leech, Raul Cabrera-Rubio, Aaron M. Walsh, Guerrino Macori, Calum J. Walsh, Wiley Barton, Laura Finnegan, Fiona Crispie, Orla O’Sullivan, Marcus J. Claesson, Paul D. Cotter

**Affiliations:** a Teagasc Food Research Centre, Fermoy, Cork, Ireland; b APC Microbiome Institute, University College Cork, Cork, Ireland; c School of Microbiology, University College Cork, Cork, Ireland; Duke University

**Keywords:** diversity, fermented, shotgun metagenomics

## Abstract

Fermented foods have been the focus of ever greater interest as a consequence of purported health benefits. Indeed, it has been suggested that consumption of these foods helps to address the negative consequences of “industrialization” of the human gut microbiota in Western society. However, as the mechanisms via which the microbes in fermented foods improve health are not understood, it is necessary to develop an understanding of the composition and functionality of the fermented-food microbiota to better harness desirable traits. Here, we considerably expand the understanding of fermented-food microbiomes by employing shotgun metagenomic sequencing to provide a comprehensive insight into the microbial composition, diversity, and functional potential (including antimicrobial resistance and carbohydrate-degrading and health-associated gene content) of a diverse range of 58 fermented foods from artisanal producers from a number of countries. Food type, i.e., dairy-, sugar-, or brine-type fermented foods, was the primary driver of microbial composition, with dairy foods found to have the lowest microbial diversity. From the combined data set, 127 high-quality metagenome-assembled genomes (MAGs), including 10 MAGs representing putatively novel species of *Acetobacter*, *Acidisphaera*, *Gluconobacter*, *Companilactobacillus*, *Leuconostoc*, and *Rouxiella*, were generated. Potential health promoting attributes were more common in fermented foods than nonfermented equivalents, with water kefirs, sauerkrauts, and kvasses containing the greatest numbers of potentially health-associated gene clusters. Ultimately, this study provides the most comprehensive insight into the microbiomes of fermented foods to date and yields novel information regarding their relative health-promoting potential.

**IMPORTANCE** Fermented foods are regaining popularity worldwide due in part to a greater appreciation of the health benefits of these foods and the associated microorganisms. Here, we use state-of-the-art approaches to explore the microbiomes of 58 of these foods, identifying the factors that drive the microbial composition of these foods and potential functional benefits associated with these populations. Food type, i.e., dairy-, sugar-, or brine-type fermented foods, was the primary driver of microbial composition, with dairy foods found to have the lowest microbial diversity and, notably, potential health promoting attributes were more common in fermented foods than nonfermented equivalents. The information provided here will provide significant opportunities for the further optimization of fermented-food production and the harnessing of their health-promoting potential.

## Abstract (precis)

Fermented foods are regaining popularity worldwide due in part to a greater appreciation of the health benefits of these foods and the associated microorganisms. Here, we use state-of-the-art approaches to explore the microbiomes of 58 of these foods, identifying the factors that drive the microbial composition of these foods and potential functional benefits associated with these populations. Food type, i.e., dairy-, sugar-, or brine-type fermented foods, was the primary driver of microbial composition, with dairy foods found to have the lowest microbial diversity and, notably, potential health promoting attributes were more common in fermented foods than nonfermented equivalents. The information provided here will provide significant opportunities for the further optimization of fermented-food production and the harnessing of their health-promoting potential.

## INTRODUCTION

Fermentation is a form of food preservation with origins that can be traced back to the Neolithic age (1). Despite recent advances in food preservation and processing, fermentation continues to be widely used as a means of preservation and is the focus of renewed interest due to increased appreciation of the organoleptic, nutritive, and—especially—health-promoting properties attributed to many fermented foods (2, 3). Indeed, various fermented foods have been shown to have enhanced attributes relative to the corresponding raw ingredients by virtue of the microbial metabolites produced (4–8), the removal of allergens (9), other desirable biological activities (10, 11), and/or the presence of microbes that have the potential to confer benefits following consumption (12, 13). Furthermore, although antibiotic use, sanitation, and food processing have greatly reduced the number of deaths due to infectious diseases, these activities have also minimized our exposure to microbes and are thought to have contributed to the “industrialization” of the human microbiome and associated increases in chronic diseases (14, 15). It has been suggested that fermented foods offer a means of safe microbial exposure to compensate for the absence/removal of desirable host microbes (15, 16). Due to these potential benefits and to an increasing appreciation that the study of these foods provides valuable fundamental insights into simple microbial communities (17, 18), developing an even greater understanding of the microbiology of these foods has the potential to be of considerable value.

Advances in high-throughput sequencing technology have revolutionized the study of microbial populations, including those present in foods. Although, to date, the vast majority of studies relating to fermented foods have employed amplicon sequencing to study bacterial and fungal composition (19–36), there have been some exceptional studies in which shotgun sequencing has been used to gain a greater insight into the taxonomy and functional potential of specific fermented foods (37–50). Despite this, studies across a broad variety of such foods using this approach have been lacking to date. Here, we address this issue by using shotgun metagenomic sequencing to investigate the microbiota of a broad range of artisanal fermented foods, including many that were previously unexplored.

## RESULTS

### Fermented-food microbiomes can be distinguished on the basis of substrate type.

**FIG 1:**
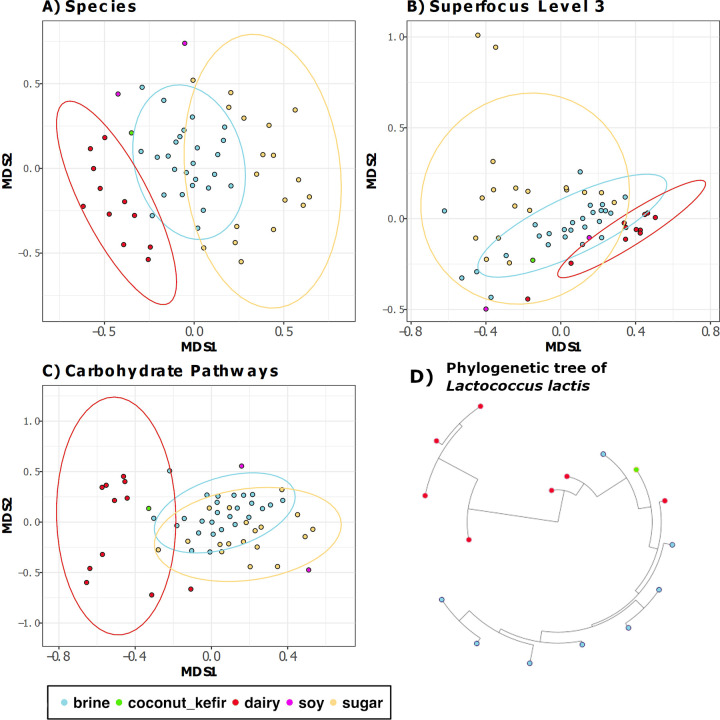
Beta diversity. (A) Nonmetric multidimensional scaling (NMDS) of Bray-Curtis distances between 58 samples, calculated for species-level composition. Samples are colored by substrate. (B) NMDS of Bray-Curtis distances between 58 samples, calculated for the Superfocus level 3 composition. Samples are colored by substrate. (C) NMDS of Bray-Curtis distances of carbohydrate pathways assigned with HUMAnN2. Samples are colored by substrate. (D) Maximum-likelihood phylogenetic tree of 16 Lactococcus lactis strains from different food samples. Strains are colored according to food substrate source. All figures show clear shifts in samples/strains by substrate.

The microbiomes of the consumable portion of 58 fermented-food samples (347,841,507 total reads; with an average of 5,997,267 reads per sample), the majority of which represented three main substrate types, i.e., dairy (such as kefir and cheese; *n* = 11), brine (such as sauerkraut and kimchi; *n* = 26), and sugar (such as kombucha and water kefir; *n* = 18), was investigated through shotgun metagenomic. Analysis of these data and other associated metadata (i.e., country of origin [“country”], specific source of product [“producer”], presence or absence of starter culture [“fermentation”], solid or liquid foods [“state”], and [“substrate”]) (Table 1), revealed that the microbiomes of these foods most significantly clustered on the basis of food substrate (Table 2 and Fig. 1). Ten characteristics of the food microbiome were defined, and differences across these characteristics were statistically examined (Table 2).

**TABLE 1:** Fermented foods and metadata^*a*^

Sample	ID	Origin	Producer	Substrate	State	Fermentation
Wagashi rind	FS00a	Benin	1	Dairy	Solid	Starter
Wagashi core	FS00b	Benin	1	Dairy	Solid	Starter
Bread kvass	FS01	Russia	2	Sugar	Liquid	Starter
Carrot kimchi	FS02	UK	2	Brine	Solid	Spontaneous
Boza	FS03	UK	2	Sugar	Liquid	Starter
Turnip	FS05	UK	2	Brine	Solid	Spontaneous
Orange	FS06	UK	2	Sugar	Solid	Spontaneous
Krauthehi (sauerkraut)	FS07	Germany	2	Brine	Solid	Spontaneous
Tepache	FS08	Mexico	2	Sugar	Liquid	Spontaneous
Ginger beer	FS09	UK	2	Sugar	Liquid	Spontaneous
Tempeh	FS10	UK	2	Soy	Solid	Starter
Cucumber	FS11	UK	2	Brine	Solid	Spontaneous
Milk kefir	FS12	UK	2	Dairy	Liquid	Starter
Water kefir	FS13	UK	2	Sugar	Liquid	Starter
Tofu chili	FS16	China	3	Soy	Solid	Spontaneous
Daikon	FS17	China	3	Brine	Solid	Spontaneous
Pickled vegetables	FS19	China	3	Brine	Solid	Spontaneous
Raw sauerkraut and juniper berries	FS22	Ireland	4	Brine	Solid	Spontaneous
Brown rice amazake	FS23	Japan	4	Brine	Solid	Spontaneous
Beetroot kvass	FS24	Ireland	5	Brine	Liquid	Starter
Kefir and fennel soup	FS25	Ireland	5	Dairy	Liquid	Starter
Mead	FS26	Ireland	5	Sugar	Liquid	Spontaneous
Sauerkraut	FS27	Ireland	5	Brine	Solid	Spontaneous
Dill dearg (sauerkraut)	FS28	Ireland	6	Brine	Solid	Spontaneous
Kimchi	FS29	Ireland	6	Brine	Solid	Spontaneous
Golden child (sauerkraut)	FS30	Ireland	6	Brine	Solid	Spontaneous
Water kefir hibiscus	FS31	Ireland	6	Sugar	Liquid	Starter
Water kefir lemon	FS32	Ireland	6	Sugar	Liquid	Starter
Water kefir ginger	FS33	Ireland	6	Sugar	Liquid	Starter
Kombucha vinegar	FS34	Ireland	6	Sugar	Liquid	Starter
Ryazhenka	FS35	Russia	7	Dairy	Liquid	Starter
Agousha	FS36	Russia	7	Dairy	Liquid	Starter
Rostagroèkport vorožnyj	FS37	Russia	7	Dairy	Solid	Starter
Ruž’a	FS38	Russia	7	Dairy	Solid	Starter
Sauerkraut	FS39	Ireland	8	Brine	Solid	Spontaneous
Kombucha	FS40	Ireland	8	Sugar	Liquid	Starter
Apple cider vinegar	FS41	Ireland	8	Sugar	Liquid	Starter
Raw milk kefir	FS42	Ireland	9	Dairy	Liquid	Starter
Pasteurized milk kefir	FS43	Ireland	9	Dairy	Liquid	Starter
Water kefir (pear, ginger, and honey)	FS44	Ireland	9	Sugar	Liquid	Starter
Water kefir (pear, ginger, and sugar)	FS45	Ireland	9	Sugar	Liquid	Starter
Dilly carrots	FS46	Ireland	10	Brine	Solid	Spontaneous
Brussels sprout kimchi	FS47	Ireland	10	Brine	Solid	Spontaneous
Kimchi	FS48	Ireland	10	Brine	Solid	Spontaneous
Garlic kraut	FS49	Ireland	10	Brine	Solid	Spontaneous
Dukkah kraut	FS50	Ireland	10	Brine	Solid	Spontaneous
Ginger sliced in 2% brine	FS51	Ireland	10	Brine	Solid	Spontaneous
Daikon radish in 2% brine	FS52	Ireland	10	Brine	Solid	Spontaneous
Okra in 2% brine	FS53	Ireland	10	Brine	Solid	Spontaneous
Tomatoes and mustard seeds in 2% brine	FS54	Ireland	10	Brine	Solid	Spontaneous
Kombucha	FS55	Ireland	10	Sugar	Liquid	Starter
Cherry water kefir	FS56	Ireland	10	Sugar	Liquid	Starter
Beet kvass	FS57	Ireland	10	Brine	Liquid	Starter
Coconut kefir	FS58	Ireland	5	Coconut_kefir	Liquid	Starter
Carrot sticks	FS59	Ireland	5	Brine	Solid	Spontaneous
Labne	FS60	Ireland	5	Dairy	Solid	Starter
Lemon and ginger fizz	FS61	Ireland	5	Sugar	Liquid	Starter
Scallion kimchi	FS62	Ireland	5	Brine	Solid	Spontaneous

a “Origin” indicates country of origin, “Producer” is a numeric code for each producer who supplied foods, “Substrate” lists the main ingredient fermented, “State” discriminates between solid and liquid foods, and “Fermentation” refers to whether a starter culture was used (starter) or not (spontaneous).

**TABLE 2:** ANOSIM results in order by descending *R* statistic^*a*^

*R* statistic	Level	Variable	*P*	*P*_adj_
0.651	Family	Type	0.001	0.008
0.551	Genus	Type	0.001	0.013
0.514	Carbs	Type	0.001	0.004
0.436	Species	Type	0.001	0.050
0.345	Superfocus level 3	Type	0.001	0.004
0.289	Superfocus level 1	Type	0.001	0.005
0.280	Phylum	Type	0.001	0.006
0.221	Carbs	Producer	0.001	0.004
0.210	Superfocus level 2	Type	0.001	0.005
0.202	Family	Fermentation	0.001	0.006
0.171	Species	Fermentation	0.001	0.017
0.169	Species	State	0.001	0.025
0.167	Family	State	0.001	0.007
0.163	AMR	Type	0.004	0.010
0.160	Species	Producer	0.003	0.008
0.154	Carbs	Fermentation	0.001	0.003
0.149	Genus	Fermentation	0.001	0.010
0.117	Superfocus level 1	State	0.002	0.006
0.111	Superfocus level 3	Fermentation	0.002	0.006
0.106	AMR	Fermentation	0.005	0.012
0.097	Genus	State	0.007	0.015
0.094	Superfocus level 3	State	0.006	0.013
0.093	Superfocus level 1	Fermentation	0.002	0.006
0.080	Superfocus level 2	Fermentation	0.006	0.014
0.076	Superfocus level 2	State	0.012	0.024
0.073	Carbs	State	0.019	0.035
0.070	Bacteriocin	State	0.018	0.035

a Only results that remained significant (*P* < 0.05) after Benjamini-Hochberg corrections (i.e., Benjamini-Hochberg adjusted *P* values [*P*_adj_]) are included here (see the full table in Data Set S1, sheet 8). AMR, antimicrobial resistance; Carbs, carbohydrates.

10.1128/mSystems.00522-20.10 DATA SET S1 (Sheet 1) Species level relative abundance of 58 foods. (Sheet 2) Superfocus level 1 relative abundance of 58 foods. (Sheet 3) Superfocus level 2 relative abundance of 58 foods. (Sheet 4) Superfocus level 3 relative abundance of 58 foods. (Sheet 5) Bacteriocin profile (in gene counts) of 58 foods. (Sheet 6) Fermented-food groups used for statistical analyses including *N* number of samples in each group. (Sheet 7) The genes that were shown in previous studies to be necessary for some probiotic attribute. These genes were then searched for in the metagenomes in this study. (Sheet 8) ANOSIM results for all 50 profiles. (Sheet 9) Table showing foods included in this study and the recipes used to make them. Some of the recipes are directly from the producer. When this information was not available, recipes were created from other online resources. Download Data Set S1, XLSX file, 1.0 MB. Copyright © 2020 Leech et al. 2020 Leech et al. This content is distributed under the terms of the Creative Commons Attribution 4.0 International license.

**FIG 2:**
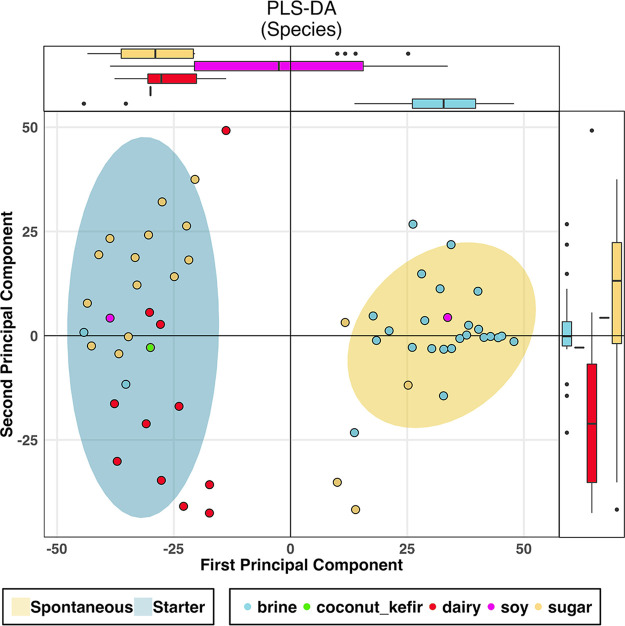
PLS-DA variance of sample clustering according to fermentation process and primary substrate. Constrained PLS-DA ordination of samples according to fermentation process illustrates that not all samples exhibit coordination of detected species composition that is dependent on the classification of the fermentation process. Samples deviating from the core fermentation-type clusters show unique compositions. PLS-DA, partial least-squares discriminant analysis. Ellipses represent confidence levels of 0.9 for the respective data. Axis plots are boxplots of the plotted data, illustrating the distribution of samples according to axis.

Taxonomy was the most distinguishing feature of the food substrates, as measured by the R statistic, supported by nonmetric multidimensional scaling (NMDS) plots and partial least-squares discriminant analysis (PLS-DA) (Fig. 1 and 2; Table 2). Substrate-related differences were greatest at the family level but were also significant at the species, genus, and phylum levels (Table 2). To further determine whether taxonomic differences at species level across substrates extend to the strain level, a further analysis of Lactococcus lactis*-*assigned reads, selected on the basis of being the species present across the greatest number of food samples, revealed that strains also phylogenetically cluster according to food substrate (Fig. 1), with samples of the same type having a lower cophenetic distance than samples of different types (*P* < 0.05). There was no clustering of L. lactis strains according to any other factor. Functional analysis revealed that substrate had the most considerable impact on the functional profile of the foods (Table 2 and Fig. 1). Carbohydrate pathways also most considerably differed across the food groups (Table 2). Indeed, of the features examined, the bacteriocin gene profile was the only characteristic that was not statistically different across the food substrates.

Three foods tested did not correspond to one of the three main food substrates, and their microbiomes were also distinct (Fig. 1). Two of these were derived from soy-based fermentations, which are known for their alkaline fermentation environment (51), and the third was a coconut kefir, i.e., a dairy kefir grain-based fermentation but of a coconut carbohydrate.

### Starter presence/absence, solid/liquid state, and producer contribute to differences in microbiota.

Although less obvious from a clustering perspective, other factors, such as starter presence/absence, solid/liquid state, and producer, were also significant drivers of microbiome differences (Fig. S1, Table 2). The presence or absence of a starter culture was associated with differences in family, species, carbohydrate, genus, Superfocus level 3 (SF3), and the antimicrobial resistance (AMR) profile of foods (in order of descending effect size), but to a lesser extent than substrate. Superfocus software assigns function to a metagenome and collapses the functions into 3 levels of specificity, with level 3 being the most specific. Solid/liquid state was significant at three taxonomic levels and all four functional profiles (three Superfocus levels and HUMAnN2 carbohydrate pathways), but again with a smaller effect size than substrate and starter status (Table 2). However, it was the only factor that was associated with significant differences across bacteriocin profiles. The specific producer of the foods was reflected by the carbohydrate-related functions and species composition, but the country of origin, in instances where a sufficiently large number of samples were sourced from a specific country, did not influence any of the factors investigated (Table 2).

10.1128/mSystems.00522-20.2 FIG S1 NMDS plots of the Bray-Curtis distances of the species in 58 fermented foods colored by substrate (A), fermentation starter or spontaneous (B), country of origin (C), producer (D), and solid or liquid foods (E). Download FIG S1, TIF file, 2.3 MB. Copyright © 2020 Leech et al. 2020 Leech et al. This content is distributed under the terms of the Creative Commons Attribution 4.0 International license.

### Microbial diversity differs between dairy foods and other food types.

**FIG 3:**
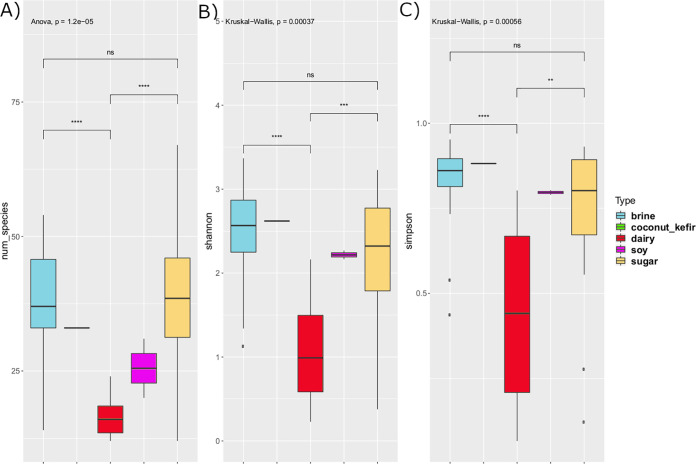
Alpha diversity by substrate. (A) Number of species (abundance >0.1%) per sample. Analysis of variance (ANOVA) was used since the data had a normal distribution. (B) Shannon index of samples. Kruskal-Wallis was used since the data were nonparametric. (C) Simpson’s diversity index of samples. Kruskal-Wallis was used since the data were nonparametric. For all three panels, pairwise tests were carried out between dairy, brine, and sugar (*t* test for parametric and Wilcoxon pairwise test for nonparametric). Coconut kefir and soy had insufficient sample sizes for pairwise comparisons.

**FIG 4:**
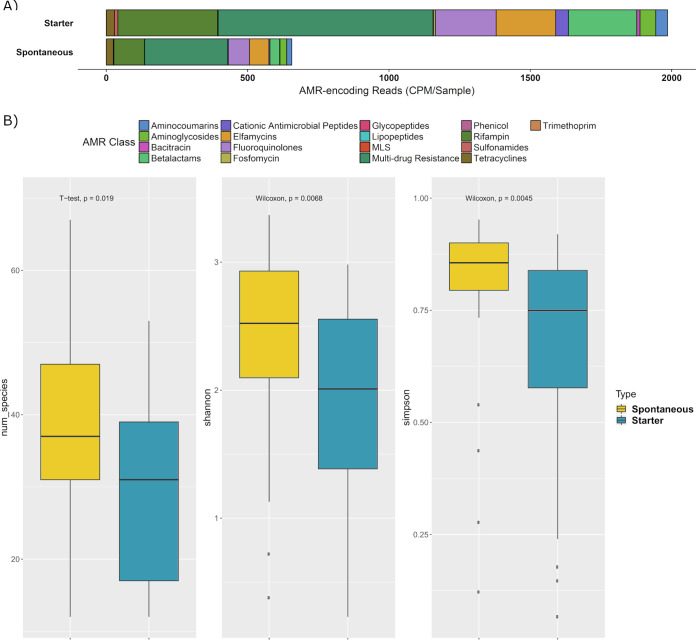
Differences by fermentation. (A) AMR profile of spontaneous fermented foods and starter culture foods. The AMR classes are normalized by counts per million per sample (CPM). (B) Alpha-diversity boxplots examined across fermentation type (spontaneous or starter). A *t* test was used for number of species since the data were parametric; a Wilcoxon test was used for the Shannon diversity index and Simpson’s index since the data were nonparametric.

Overall, 476 unique species, present at above 0.1% relative abundance, were assigned to the 58 foods, 301 different species of which were detected in brine foods, 242 in sugar foods, and 70 in dairy foods. This corresponded to an average of 11.5, 13.5, and 6.4 different species per sample for brine, sugar, and dairy foods, respectively. In line with these results, alpha-diversity analyses demonstrated that the microbiomes of dairy-based fermented foods had significantly lower alpha diversity than those of either brine or sugar foods (Fig. 3), which did not significantly differ from one another. It was also evident that, as expected, the alpha diversity of spontaneously fermented foods was significantly higher than those produced using starter cultures (Fig. 4). Across the specific foods, a spontaneously fermented orange preserve contained the highest number of species (*n* = 67), while a sample of tepache, a slightly alcoholic spontaneously fermented drink from Mexico, contained the lowest number of observed species (*n* = 12).

### Lactic acid bacteria dominate brine foods.

The brine-type foods tested comprised 26 plant substrate-derived foods fermented in a saline solution. Unlike both dairy- and sugar-type fermented foods, the majority of the brine-based foods undergo a spontaneous fermentation and therefore rely on fermentation by autochthonous microbes (52). Brine foods mostly contained bacteria, with fungal assignment being 3.9% of the phylum level relative abundance. Archaea accounted for less than 0.5%. Among brine-type foods, *Lactobacillus* was the most abundant genus, comprising 46.8% of all reads assigned at the genus level. *Lactiplantibacillus plantarum* was the most abundant species (9.6% relative abundance on average), followed by L. brevis (7.9%), *L. mucosae* (4.7%), *L. xianfangensis* (4.1%), and *L. sakei* (3%). Leuconostoc mesenteroides (4.7%) and Pediococcus parvulus (4.3%) were also present in significant quantities. Across the brine-type foods, *Bifidobacteriaceae* were detected at a relative abundance of 1.6%. At the species level, 0.8% of species were assigned as Bifidobacterium longum, and 0.01% were assigned to *B. breve*. No other bifidobacteria were assigned at the species level.

The seven sauerkraut samples and five kimchi samples analyzed contained many of the genera regarded as being typical of these foods, such as *Lactobacillus*, *Leuconostoc*, the yeast *Pichia*, *Rahnella*, and the yeast *Kazachstania* (21, 24). Some more unusual species were found at low abundance, including *Perkinsus marinus* (0.33%), an oyster pathogen, which was detected in a scallion kimchi sample and possibly originated from the shrimp paste used in the manufacture of this kimchi. Other brine-type fermented foods were investigated using this approach for the first time. These included a lacto-fermented cucumber, fermented green tomatoes, and beet kvass, the latter being classified as a brine-type food since, unlike other typical kvass, this product contained large quantities of salt and no added sugar. These foods contained many plant-associated taxa, including large proportions of *Pseudomonas*, *Lactobacillus*, and *Pediococcus*. Brown rice amazake was particularly notable by virtue of containing a high relative abundance of B. longum (7.3%). A corresponding metagenome assembled genome (MAG) was recovered and found to be a 98.8% match with a previously sequenced B. longum E18 genome. In total, B. longum was detected across 4 of the 7 sauerkraut samples, 3 of the 5 kimchi samples, and 9 of the 13 other brine samples. The microbiota of other vegetables fermented in brine (Table 1) is described in greater depth in Data Set S1, sheet 1, in the supplemental material.

From a functional potential perspective, 18.4% of Superfocus level 1 (SF1) functions within the brine food microbiome were predicted to relate to carbohydrate metabolism. When functional pathways were investigated at a deeper level, xylose utilization (0.6%, SF3), fermentation (1.4%, SF2), and response to osmotic stress (1%, SF2) were among the most common functionalities (Data Set S1, sheets 2 to 4). A complete list of the relative abundances of the Superfocus pathways, for all foods, can be found in Data Set S1, sheets 2 to 4.

### The microbiota composition of dairy foods is more homogeneous than that of other fermented foods.

Eleven dairy-type fermented foods were studied. Information supplied by the producers established that all of these foods were produced through the use of starter cultures to initiate fermentation, thus likely contributing to their reduced diversity relative to other foods (21). Dairy foods contained the least eukaryote-assigned reads, with 1.6% of the phylum-level relative abundance assigned to fungal groups. *Firmicutes* (89.2%) and *Proteobacteria* (7.9%) dominated. L. lactis dominated at the species level, corresponding to, on average, 44.8% of relative abundance and was present at a relative abundance at or above 90% in three of the dairy foods, all of which were kefir or kefir-type foods. The next most abundant species was Streptococcus thermophilus (16%), followed by *S. infantarius* (5.7%), the yeast Kluyveromyces marxianus (3.7%), Escherichia coli (3.5%), Lactococcus raffinolactis (3%), and *L*. *mesenteroides* (2.9%). It is notable that viruses [including (pro)phage] also made up a significant portion of the dairy food microbiota (7.8%).

Kefir composition, including that of agousha, a Russian beverage akin to kefir, was consistent with previous studies (25, 53, 54), although some taxa not previously associated with kefir, such as Bifidobacterium mongoliense, were detected. Two samples of kefir were fermented with the same kefir grain but differed in that one was made with pasteurized milk (FS43), while the other was made with unpasteurized milk (FS42) (Table 1). While there were a number of species found in only one of these two samples, only one, Pseudomonas helleri, found in pasteurized milk, was present at above 1% abundance (3%). Profiling of a samples of wagashi, a cheese from Benin, for the first time revealed that the core and the rind had a similar taxonomic composition, and included *S. infantarius*, K. marxianus, and a worrying abundance of Escherichia coli. Ryazhenka, a fermented baked milk product from Russia, was enriched with S. thermophilus (33.2%). Other species, such as *S. macedonius* (2.8%), were also present, but the most striking feature of this food was the 54% assignment to viruses. Kraken (55) was used to investigate the viral component in more detail. A total of 97% of these reads were assigned to the order *Caudovirales* and, in turn, 99.9% of these *Caudovirales* were assigned as *Streptococcus phage*, with *Streptococcus* phage DT1 (57%) being the most abundant. Ruž’a, a Russian cheese, had a large relative abundance of L. lactis (50.1%), followed by *L. mesenteroides* (25%) and *L. raffinolactis* (15.9%). Another Russian sample, rostagroèkport vorožnyj, a quark-like fermented snack, was high in S. thermophilus (50.5%). Irish labne consisted mainly of S. thermophilus (86%) but also contained *Lacticaseibacillus paracasei* (3.2%) and *L. casei* (2.3%).

At a functional level, carbohydrate metabolism (16.7%) was the most abundant SF1 pathway in fermented dairy. SF2 results highlighted the presence of genes with homology to those encoding resistance to antibiotics and the production of toxic compounds (2.8% of the reads). Several of the most abundant SF3 pathways in dairy foods had phage related functions, including the most abundant function, i.e., phage head and packaging (3.2%).

### Sugar foods are dominated by *Acetobacteraceae*.

Eighteen sugar-type fermented foods were assessed, including fermented fruit, kombucha, and water kefir. Some of these foods, such as kombucha, kvass, and water kefir, contained large quantities of added table sugar, whereas the substrates used for the production of fermented orange or mead, honey, and water, had naturally high levels of sugar. Furthermore, although these foods were all assigned to the “sugar foods” category (Table 1), they encompassed a wide variety of raw ingredients and fermentation methods, including examples of both spontaneous and starter-type fermentations.

Sugar based fermentations contained the highest abundance of reads assigned to fungi, with 19.7% of phylum level reads assigned to *Ascomycota*. Similar to the other foods, *Proteobacteria* (48.9%) and *Firmicutes* (28.2%) dominated. Sugar foods contained many species previously associated with alcohol-generating fermentations, such as the yeasts *Saccharomyces eubayanus* (2.7%), *Brettanomyces bruxellensis* (5.2%), *Hanseniaspora valbyensis* (9.3%), and the bacterial species Oenococcus oeni (5%). Many of the other species were well-known kombucha-associated species such as Gluconobacter oxydans (5%), Acetobacter cerevisiae (2.5%), and Komagataeibacter rhaeticus (2%). At the species level, *H. valbyensis* was the most abundant (9.3% average abundance). However, this reflects very high abundance in specific instances, e.g., the relative abundance in mead was 93.7%, whereas this species was not detected in 10 of the other 18 sugar-type fermented foods. *Lactobacillus* was the most abundant genus (25.8%), but its abundance was lower than that found for dairy and brine foods. Within this genus, *L. mali* (7.6%) and *L. plantarum* (5.3%) were the most common species. *Acetobacter* was the next most abundant genus (10.9%), and its distribution, along with other members of the *Acetobacteraceae*, made it the most abundant family (33.3%).

Among specific sugar food types, seven samples of water kefir were analyzed, and typical water kefir-associated taxa, including *Kluyvera*, *Gluconobacter*, *Brettanomyces*, *Acetobacter*, and *Lactobacillus* (27), were found. In addition, Ethanoligenens harbinense, a species previously found in the wastewater that results from molasses production (56), was present in three of the water kefir samples. Two kombucha samples and a kombucha vinegar sample were examined. Typical kombucha microorganisms were identified (22, 23, 26). However, while the genera were similar, the abundance and type of species differed, e.g., *Komagataeibacter* and *Acetobacter*, were present in both kombucha samples, but one sample contained 13.7% *K. xylinus* and 5.2% *A. okinawensis*, while the other sample had 34.2% *K. rhaeticus* and 4.1% *A. senegalensis* (see Data Set S1, sheet 1). The microbiota of tepache, a slightly alcoholic Mexican fermented beverage, was investigated through the use of shotgun sequencing for the first time. Tepache contained the lowest number of species of all foods, consisting mainly *of L. plantarum* (85%), *Levilactobacillus brevis* (4.6%), and Acetobacter syzygii (3.6%). Mead, produced using autochthonous microbes present in honey, contained four different species of the yeast *Hanseniaspora*, including the aforementioned *H. valbyensis*. *Hanseniaspora* has not been described in mead previously, with *Saccharomyces* generally being the most common genus (57), and is widely used for industrial-scale mead production (58). The mead sample was also notable by virtue of generating by far the highest relative abundance of reads assigned to eukaryotes (>96%). In addition to *Hanseniaspora*, Zygosaccharomyces rouxii, Torulaspora delbrueckii, Saccharomyces cerevisiae, and a rare yeast, *Saitoella complicate*, were the other fungal species identified. The microbiomes of boza, orange preserve, apple cider vinegar, ginger beer, lemon and ginger fizz, bread kvass, and beet kvass are also presented in Data Set S1, sheet 1.

The most abundant SF1 function found in sugar foods was carbohydrate metabolism (14.5%). Resistance to antibiotics and toxic compounds (3.8%) and osmotic stress (1%) were the most common SF2 functions, while analysis of SF3 pathways highlighted the frequency of several pathways involved in the synthesis of amino acids, such as both methionine (0.79%) and purine (0.68%) biosynthesis.

### The fermented-food resistome differs according to food and fermentation type.

**FIG 5:**
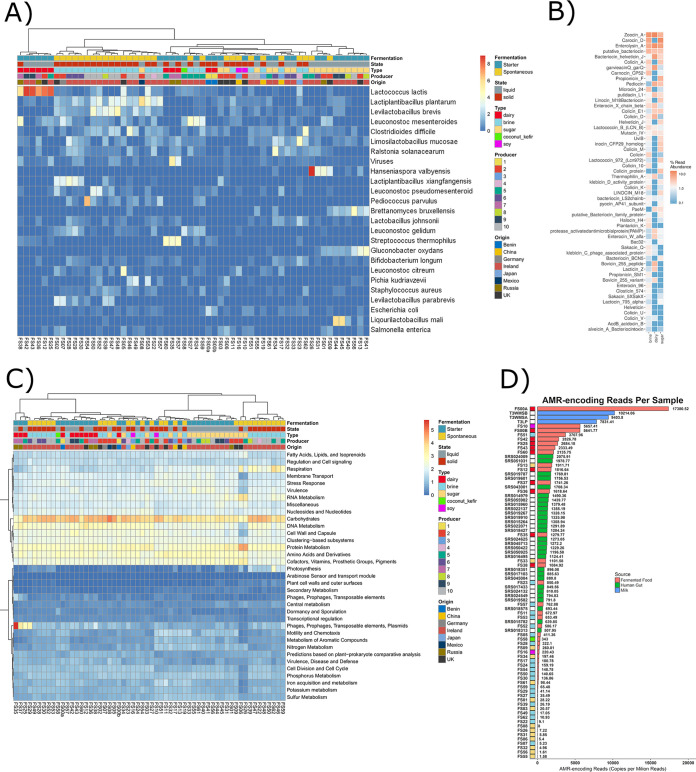
Descriptive plots. (A) Heatmap showing the square root of the relative abundance of the top 25 species across all foods. Metadata categories are shown along the top *x* axis. Both rows and columns are clustered according to similarity. (B) Heatmap showing the relative abundance of the bacteriocin profile binned according to food substrate. (C) Heatmap showing the square root of the relative abundance of the Superfocus level 1 pathways. (D) Antimicrobial resistance (AMR) genes in CPM per food (pink), per milk sample (blue), and per human sample (green). Thirteen of the sixteen milk samples and nine fermented-food samples are not shown since no AMR genes were detected in these samples. Metadata for the food substrate are indicated by the boxes on the left of the CPM bars.

Large variability in both the counts per million of antimicrobial genes (CPM) and of antimicrobial resistance (AMR) class were apparent across the different foods, with AMR profiles significantly differing across substrate and in line with the presence/absence of a starter (Fig. 4A, Fig. 5D, and Table 2). Dairy had an average of 3,686 CPM per sample, brine had 426 CPM, and sugar had 261 CPM. However, the core and the rind of wagashi inflated the dairy results and, if these are excluded, the average CPM for dairy foods dropped considerably to 1,947.

With respect to specific AMR classes, multidrug resistance was the most commonly assigned gene category across all three food substrates, corresponding to 2,422, 293, and 133 CPM per sample on average for dairy-, brine-, and sugar-type foods, respectively. Beta-lactam resistance genes were the next most common class in dairy (718 CPM) and sugar (101 CPM) foods, while tetracycline resistance genes were the second most numerous category of AMR genes in brine (45 CPM). It was also noted that a 5-fold-higher abundance of AMR genes occurred in starter culture fermentations relative to spontaneous fermentations. Multidrug resistance genes again dominated, corresponding to 1,326 CPM for starter cultures and 236 CPM for spontaneous fermentations. Beta-lactam resistance genes were the next highest in foods containing starter cultures (428 CPM), whereas tetracycline resistance genes were next highest in spontaneously fermented foods (48 CPM). The high CPM for both dairy and starter containing foods are consistent with the fact that dairy foods were those for which starters were most extensively used. When gene distribution was investigated from the perspective of specific food substrates, the wagashi cheese rind was found to have the highest CPM, i.e., 17,381, with tempeh being next highest at 5,657 CPM. AMR genes counts in kombucha and water kefirs were generally low, and no known AMR genes were identified in 9 of the 58 foods, i.e., 1 kombucha, 2 water kefirs, 3 kimchi, 1 pickled carrot, 1 pickled vegetable, and 1 apple cider vinegar. Of the nine fermented foods for which no AMR genes were assigned, four were sugar-type (including two water kefirs) and five were brine-type (including three kimchis). It is notable that very few AMR genes were assigned in the two other kimchis studied (<42 CPM), while across the five other water kefir samples, three contained very few AMR genes (<6 CPM) but two had relatively high counts (>1,000 CPM). Across the two samples of kombucha, one did not contain assigned AMR genes, while the other contained 1.6 CPM.

To provide context, the frequency with which AMR genes are detected in fermented foods was compared to that across human stool samples and unfermented milk samples (Fig. 5D). Milk samples (*n* = 16) were from various stages of processing to produce skim milk powder, including unpasteurized milk silos and dry milk powders. Although a subset of three milk (unpasteurized) samples had relatively high AMR CPM, milk samples generally had lower AMR CPM than did fermented-food or human gut samples (*P* > 0.01). In contrast, human gut samples (29 random stool samples from the Human Gut Microbiome Project [59]) had significantly more AMR CPM than fermented foods and milk (*P* > 0.01). However, eight fermented foods had particularly high CPM of AMR genes. These eight foods were the two wagashi cheese samples, tempeh, fermented ginger, three milk kefirs, and labne. Of these eight foods, six were dairy, and seven were starter-generated foods. For a further 12 foods, the AMR CPM were similar to those for human samples, whereas 38 foods had AMR CPM that were lower than those for human samples.

### The presence of putative health promoting genes differs markedly across fermented foods but exceeds that of nonfermented foods.

Bacteriocins are ribosomally synthesized antimicrobial peptides, many producers of which have been sourced from fermented foods. The bacteriocin-producing potential across the 58 fermented-food samples was investigated, with 55 putative bacteriocin-encoding gene clusters being assigned across 54 of the foods (no gene clusters identified in 4 samples (Data Set S1, sheet 5). Zoocin A- and enterolysin A-like gene clusters were highly abundant across all three fermented-food substrates. Clusters corresponding to another bacteriolysin subclass, the helveticin J-like proteins, were more frequently detected in dairy and sugar-type foods than in brine-type foods (Fig. 3B). Carocin D- and colicin A-like clusters had a high abundance in brine and sugar, but not dairy, foods. As noted above, there was a significant difference in the distribution of bacteriocins between solid and liquid food types (Table 2), with liquid foods having a higher relative abundance of helveticin J, propioncin F-like and pediocin clusters, and solid foods having more carnocin CP52-like and microsin 24-like clusters. Examining the pediocin sequences in more detail revealed homology with *pedA* and *pedB*.

**FIG 6:**
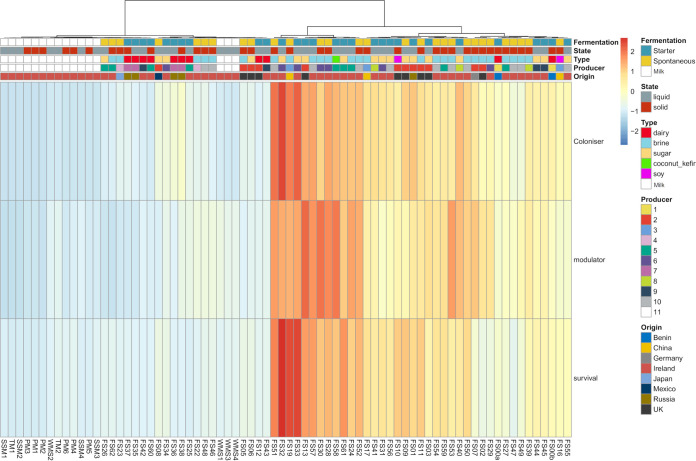
Heatmap showing the presence of potentially health-associated gene clusters (PHAGCs) across all 58 foods and 16 unfermented milk samples. Gene clusters are binned as potentially inferring an ability of the metagenome to colonize the gastrointestinal tract, survive transit to the gut, and modulate the host phenotype. Each row is normalized across all samples, thus only comparing foods to one another.

Given that bacteriocin production is regarded as a probiotic trait, these findings prompted an investigation of other potentially health-associated gene clusters (PHAGCs) within these fermented-food microbiomes. PHAGCs were divided into three broad categories. Gene clusters binned as “survival” are genes that were shown to be important for surviving the low pH of the stomach or the bile salts of the small intestine (60). Gene clusters binned as “colonization” are genes that were shown to be vital for colonizing the gut microbiome. These included genes responsible for surface proteins and exopolysaccharide production. “Modulation” gene clusters were all of the other potentially health promoting gene clusters that did not fit the previous two bins. These genes were shown to affect the host phenotype in other ways, such as stimulating the host immune system in the case of d-phenyl-lactic acid (13) or the production of γ-aminobutyric acid (GABA) (61, 62). The majority of these PHAGCs genes are based on studies reviewed in reference 60). Shotgun metagenomic data from nonfermented foods, i.e., unpasteurized whole milk, pasteurized skimmed milk, and milk powder, was used for comparative purposes. In general, the fermented foods contained considerably more PHAGCs than the nonfermented substrates. Among the fermented foods, a larger number of PHAGCs were found in brine- and sugar-type foods than in dairy foods, with several water kefirs, sauerkrauts, beet kvasses, and one kombucha being the foods with highest levels of PHAGCs (Fig. 6). With respect to the individual PHAGC subcategories, all fermented foods contained more colonization-type PHAGCs than the nonfermented controls. In the case of the modulation and survival clusters, the number of PHAGCs in some fermented foods, such as scallion kimchi, labne, agousha, and mead, were no greater than those in the nonfermented foods.

### Metagenomic assembly reveals 10 putative new species.

**FIG 7:**
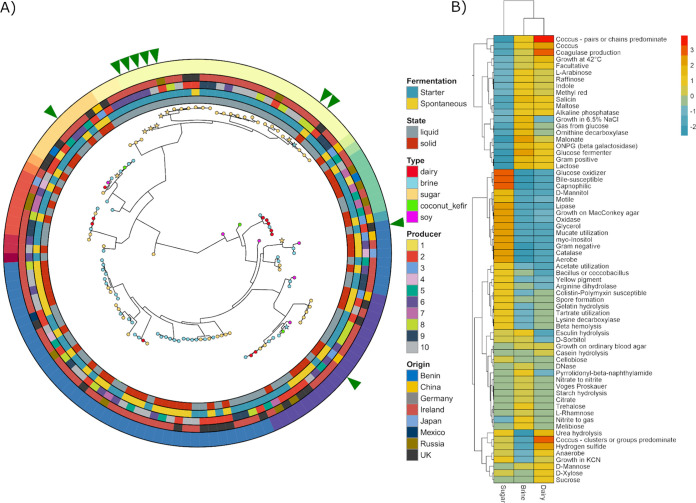
Metagenome assembled genomes. (A) Phylogenetic tree of the 127 high-quality MAGs, with outer rings showing the metadata for the food. The green arrows indicate which MAGs are potentially novel species. (B) Predicted phenotypes of the 127 MAGs concatenated into their respective substrate. Both rows and columns are clustered according to similarity.

Metagenome assembled genomes (MAGs) were assembled from the reads and quality checked. A total of 443 MAGs were assembled in total, with 127 genomes above 80% completeness and having less than 10% contamination (Fig. 7A). Traitar (63) was used to predict the growth phenotypes of the 127 MAGs. The outputs were concatenated into a single output for each food substrate (Fig. 7B) and provided intuitive results, such as a high correlation between lactose utilization and dairy foods and high glucose oxidation potential in sugar food microbiomes. Consilience between the Traitar and taxonomic output is supported by the abundance of L. lactis in dairy and brine samples. FastANI (64) was used to assign taxonomy and to assess novelty and established that 10 of these MAGs had an <95% identity to known NCBI prokaryote genomes. Seven of these potentially novel MAGs are acetic acid bacteria, two are lactic acid bacteria, and one belongs to the family *Yersiniaceae* (Table 3). The highest identity match for three of the novel MAGs was Acidisphaera rubrifaciens. All three of these MAGs came from water kefir. The four remaining acetic acid bacteria were best matched with Acetobacter aceti (MAG from water kefir), Gluconobacter cerinus (MAG from bread kvass), and Acetobacter malorum (MAGs from rostagroèkport vorožnyj and apple cider vinegar). The two novel LABs were best matched with *Leuconostoc gelidium* (sauerkraut MAG) and *Companilactobacillus kimchiensis* (boza MAG). The final novel MAG, from the water kefir microbiome, most closely resembled Rouxiella chamberiensis.

**TABLE 3:** Putatively novel MAGs with FastANI identity scores to the closest genome in the NCBI database

Food	Sample	Closest NCBI match	% Identity
Bread kvass	FS01	Gluconobacter cerinus	93.4228
Raw milk kefir	FS41	Acetobacter malorum	86.3852
Sauerkraut	FS39	Acetobacter malorum	85.9458
Boza	FS03	Companilactobacillus kimchiensis	82.2453
Water kefir lemon	FS32	Rouxiella chamberiensis	81.3335
Golden child (sauerkraut)	FS30	Leuconostoc gelidum subsp. gasicomitatum	81.0244
Cherry water kefir	FS56	Acetobacter aceti ATCC 23746	78.5186
Water kefir hibiscus	FS31	Acidisphaera rubrifaciens HS-AP3	78.4976
Water kefir ginger	FS33	Acidisphaera rubrifaciens HS-AP3	78.475
Water kefir lemon	FS32	Acidisphaera rubrifaciens HS-AP3	78.0727

## DISCUSSION

The practice of fermenting foods can be traced back over many millennia (65). Recently, shifts in consumer preference have resulted in a renewed interest in fermented foods, with the associated global market estimated to reach $40 billion USD by 2024 (66). The development of a better understanding of the microbial composition and functional potential of these foods provides an insight into features that are common among, and different between, fermented foods and ascertain potential roles of individual species, including novel species and strains. Importantly, the taxonomic resolution of shotgun metagenomics allows strain level identification of the microbiome but also facilitates an assessment of functional profile, bacteriocin and AMR gene distribution, determination of PHAGCs, the assembly of MAGs, and the determination of predicted phenotypes. It is important to note that samples were not replicated in order to facilitate the inclusion of a larger overall number of fermented foods. Although this limits the conclusions that can be made for specific foods, a number of notable observations were made across broader fermented-food types. Furthermore, since over 5,000 varieties of fermented foods exist around the world, often with multiple varieties of each of these foods, it was not possible to represent all fermented foods. For this reason, this study has focused on a subset of artisanal fermented foods that are easy to produce at home, and thus generalizations relating to specific substrates are limited to the samples examined here.

Fermentation substrate is the strongest driver of the composition and functional potential of the microbiomes of fermented foods. The type of nutrients available to the microbes determined the diversity within each food to the greatest extent. The biggest effect of substrate was found between the families present in each food substrate, with *Lactobacillaceae* (linear discriminant analysis [LDA] = 5.68) most persistent in brine foods, *Streptococcaceae* (LDA = 5.92) in dairy foods, and *Acetobacteraceae* (LDA = 5.5) in sugar-based foods (Fig. S2 to S8). The different substrates impose functional requirements on the microbes, such as a necessity for osmotic stress tolerance in both brine- and sugar-type foods. While the study focused on substrates that are used commonly for small-scale fermented-food production, including those made most easily in the home, it will be interesting in the future to extend the analysis to other important fermentation substrates such as meat, fish, and grains. The analyses here reflect the composition of the foods typically consumed and not the starter cultures used to produce these foods or the intermediary microbiomes that eventually produce the end product.

10.1128/mSystems.00522-20.3 FIG S2 LDA plots reveal the attributes driving the differences between the food substrates at the species level. Download FIG S2, TIF file, 2.1 MB. Copyright © 2020 Leech et al. 2020 Leech et al. This content is distributed under the terms of the Creative Commons Attribution 4.0 International license.

10.1128/mSystems.00522-20.4 FIG S3 LDA plots reveal the attributes driving the differences between the food substrates at the genus level. Download FIG S3, TIF file, 2.0 MB. Copyright © 2020 Leech et al. 2020 Leech et al. This content is distributed under the terms of the Creative Commons Attribution 4.0 International license.

10.1128/mSystems.00522-20.5 FIG S4 LDA plots reveal the attributes driving the differences between the food substrates at family level. Download FIG S4, TIF file, 2.0 MB. Copyright © 2020 Leech et al. 2020 Leech et al. This content is distributed under the terms of the Creative Commons Attribution 4.0 International license.

10.1128/mSystems.00522-20.6 FIG S5 LDA plots reveal the attributes driving the differences between the food substrates at the phylum level. Download FIG S5, TIF file, 2.0 MB. Copyright © 2020 Leech et al. 2020 Leech et al. This content is distributed under the terms of the Creative Commons Attribution 4.0 International license.

10.1128/mSystems.00522-20.7 FIG S6 LDA plots reveal the attributes driving the differences between the food substrates at Superfocus level 1. Download FIG S6, TIF file, 2.0 MB. Copyright © 2020 Leech et al. 2020 Leech et al. This content is distributed under the terms of the Creative Commons Attribution 4.0 International license.

10.1128/mSystems.00522-20.8 FIG S7 LDA plots reveal the attributes driving the differences between the food substrates at Superfocus level 2. Download FIG S7, TIF file, 2.0 MB. Copyright © 2020 Leech et al. 2020 Leech et al. This content is distributed under the terms of the Creative Commons Attribution 4.0 International license.

10.1128/mSystems.00522-20.9 FIG S8 LDA plots reveal the attributes driving the differences between the food substrates at Superfocus level 3. Download FIG S8, TIF file, 2.0 MB. Copyright © 2020 Leech et al. 2020 Leech et al. This content is distributed under the terms of the Creative Commons Attribution 4.0 International license.

Other factors, such as the presence or absence of a starter culture, also contributed to differences in that starter culture-derived foods had the lowest alpha diversity, likely a result of adding a community of specialist microbes to the food that have been selected to outcompete the autochthonous microbes. The similar microbiome profiles of two kefir samples made from the same starter, but using raw or pasteurized milk, respectively, highlight this point. The differences in diversity between solid and liquid foods is likely due to the selective pressures of mobility, nutrient availability (in a homogenous liquid compared to a less homogenous solid food), and moisture content in solid foods compared to liquid foods. Unsurprisingly given the diversity of fermented foods, the country of origin did not significantly influence any of the characteristics examined. Outside of composition and top-level functionalities, other traits did vary in line with other categories, in that the bacteriocin gene cluster profile differed significantly across solid and liquid foods, and AMR-encoding genes differed across food substrate and between spontaneous and starter-type fermentations. It is unclear why bacteriocin gene clusters differed across solid and liquid foods, but perhaps the matrices of solid foods require different ecological tools for competitive advantage than liquid substrates.

Analysis revealed that the microbiomes of starter culture-type fermentations contain more assigned AMR-associated genes. However, this difference could represent the more extensive characterization of starter culture microbes and their associated genomes and AMR profiles, leading to better assignment of AMR genes from starter cultures strains than those involved in spontaneous fermentations. In comparison with human gut metagenomes, the majority of the fermented foods had a lower AMR CPM. Of the eight foods with higher AMR CPM, only three stood out as having considerably higher CPM; two were subsamples of the same food, i.e., wagashi cheese. In contrast, kimchi and kombucha samples were notable by virtue of either lacking detectable AMR genes or having very low CPM. Kimchi shared many taxa with other brine-type foods, so the differences observed may reflect strain-level differences. Comparisons with the unfermented milk samples showed that fermented foods have less AMR CPM than raw milk but more than pasteurized milk and its biproducts. Metagenomic sequencing of a larger collection of these fermented foods, coupled with antibiotic resistance assessments of isolated strains, will be necessary to determine how representative these results are.

Bacteriocin production is regarded as a probiotic trait. These peptides and, in the case of bacteriolysins, proteins are thought to be produced by bacteria to gain a competitive advantage over other taxa, typically those occupying the same environmental niche. Bacteriocin production can contribute to the quality and safety of foods through the removal of spoilage and pathogenic bacteria, but bacteriocin production *in situ* in the gut can also enable the producing bacteria to become established, compete against undesirable taxa, and contribute to host-microbe dialogue (67, 68). The bacteriocin profile did not differ according to food substrate, with zoocin A- and enterolysin A-like genes being most abundant across all food substrates. However, the bacteriocin-associated genes present in solid and liquid foods differed significantly from one another in that liquid foods were enriched with pediocin-like genes. After a further analysis of the pediocin sequences, homology with *pedA* and *pedB*, required for the production of pediocin AcH/PA-1, was apparent. These bacteriocins are best known for their strong antilisterial effects (69). Pediocin AcH/PA-1 has also been shown to be active against enterococci and staphylococci (70), and the presence of these genes potentially adds to the safety of these foods and their potential to be health promoting. Solid foods had a higher abundance of carnocin CP52-like bacteriocins, which are known for activity against *Listeria* and *Enterococcus*, again potentially adding to the safety of these foods (71).

Across a broader range of PHAGCs, it was apparent that these gene clusters were more common in fermented than in nonfermented foods. Sugar and brine foods were found to contain the highest levels of PHAGCs. Microbes in sugar-type foods generally must persist in low-pH environments, with some kombucha fermentations dropping to as low as pH 3 (72). In contrast, although also somewhat acidic, a milk kefir fermentation is regarded as complete when the pH reaches 4.5 (73), while the pH of most cheeses is between pH 5.1 and 5.9. Many of the sugar foods also contained colonization-associated PHAGCs. It was also noted that brine-type foods had the highest abundance of *Lactobacillaceae*, specific representatives of which have been exploited for their probiotic activity. A combination of these various factors likely contributes to the higher abundance of PHAGCs in both of these foods relative to dairy foods. However, even within the respective food substrate groups, the PHAGCs present varied considerably, with foods such as water kefirs, sauerkrauts, pickled veg, ginger, kvass, and kombucha being enriched in PHAGCs. These foods all contained colonization and survival PHAGCs at a higher frequency, e.g., glycotransferases for colonization in kombucha and pickled veg, and bile salt metabolism genes in water kefir and fermented sliced ginger. d-Lactate dehydrogenase pathways were consistently identified in these foods but were absent from other such as scallion kimchi, carrot sticks, and agousha. This observation is notable as d-lactate dehydrogenase is the enzyme responsible for producing d-phenyl-lactic acid (d-PLA), a metabolite known to modulate the host immune system (13). Glutamate decarboxylase, which converts glutamate into γ-aminobutyric acid (GABA), was present in some (kombucha, kvass, coconut kefir, and some water kefir samples), but not all, PHAGC-enriched foods. GABA is a well-known modulator of mood (74), while this enzymatic reaction also consumes protons and thus contributes to acid resistance (75). Although *in vivo* studies are required to directly examine the health benefits of specific fermented foods, these insights can undoubtedly help to identify foods and strains that are more likely to be health promoting, facilitate the production of fermented foods optimized for health promotion, and direct the experimental design of human intervention studies.

Finally, this study discovered 127 high quality MAGs, of which 10 are putative novel species. Three putative new *Acetobacter* species from water kefir, milk kefir, and sauerkraut, a *Gluconobacter* from bread kvass, a *Leuconostoc* from sauerkraut, and a *Companilactobacillus* from boza were assembled from the shotgun data. While these species are apparently novel, the corresponding genera are found in fermented foods at a high frequency. However, two MAGs representing genera that have not been found in fermented foods before were assembled, i.e., a *Rouxiella* species and three *Acidisphaera* species, all from water kefir samples. Rouxiella chamberiensis and Acidisphaera rubrifaciens are the only previously known members of their respective genera. Rouxiella chamberiensis was isolated from parenteral nutrition bags and has been shown to ferment d-glucose but not sucrose (76) and Acidisphaera rubrifaciens has been found in acidic hot springs and mine drainage systems and, like many of the other sugar taxa, is acidophilic (77). The assembly of these and other MAGs in the future will contribute toward the building of fermented food, and other food, microbe databases, equivalent to those available for the more complex human gut microbiome (78), to enable the more accurate and rapid identification of food microbes. Such databases will be key in the application of metagenomics-based approaches on a widespread basis by the food industry.

Overall, this study combines many novel insights into fermented-food microbiomes. First, the taxonomic composition of the 58 foods has been described, including many foods that have not been described using next generation sequencing (NGS) previously. Second, the functional profile of these foods has been characterized and, like the taxonomic profile, highlights the differences between starting material and microbial composition. Importantly, given the current interest in fermented foods as a healthy food choice and the role diet plays in modulating the gut microbiome, the health-promoting potential of the microbes in these various foods has been explored. Finally, genomes, including potentially novel taxa, were assembled from these foods and will contribute to the better assignment of reads from fermented food, and indeed broader food chain microbiome studies, in the future.

## MATERIALS AND METHODS

A total of 58 samples of fermented foods were collected from various artisanal producers (Table 1). Foods were sampled when they were ready for consumption. In all cases, the edible portion of the fermentation was sampled. For example, for foods such as milk kefir or kombucha, the starter grain or SCOBY (symbiotic colony of bacteria and yeast), respectively, were not sampled, but rather sampling focused on the liquid portion of these products that is consumed. Portions (5 g) of foods were placed in a stomacher bag. Then, 50 ml of sterile Maximum Recovery Diluent (MRD) was added to the bag. The contents were homogenized in a stomacher (BagMixer 400; Interscience) for 20 min. After this step, both solid and liquid foods were extracted using the same method. Next, 50 ml of the homogenized solution was centrifuged at 10,000 rpm, at room temperature, for 10 min. The supernatant was discarded. The pellet was resuspended in 550 μl of SL buffer in a 2-ml tube (SL buffer from the GeneAll kit described below). Then, 33 μl of proteinase K was added to the tube, followed by incubation at 55°C for 30 min. The solution was then transferred to a bead-beating tube and placed in a Qiagen TissueLyser II for 10 min at 20/s. The GeneAll Exgene extraction protocol in step 4 was then followed until the final elution step; 30 μl of elution buffer (EB) was used here instead of the 50 μl suggested in the protocol.

### Sequencing.

Library preparation was carried out according to the Nextera XT protocol (Illumina) (79). DNA was quantified by using a Qubit high-sensitivity dsDNA assay. The final library quality was assessed by using Agilent high-sensitivity DNA chromatin immunoprecipitation, and quantification was done by qPCR using a KAPA library quantification kit (Illumina; Roche). Sequencing was carried out on the NextSeq500 using a 300-cycle High Output v2 kit.

### Bioinformatics.

A total of 347,841,507 reads were obtained from the Nextseq sequencing run in the form of Bcl files, which were converted to fastq format using bcl2fastq software. Quality trimming was performed using the trimBWAstyle.usingBAM.pl script. Using Picard (https://github.com/broadinstitute/picard), fastq was converted to Sam format. Picard was also used to remove duplicates. The sequences were then quality checked and trimmed using the trimBWAstyle.usingBam.pl script from the Bioinformatics Core at UC Davis Genome Center (https://github.com/genome/genome/blob/master/lib/perl/Genome/Site/TGI/Hmp/HmpSraProcess/trimBWAstyle.usingBam.pl). Host reads accounted for >8% of the reads. They were not removed since reference genomes were not available for all food substrates. All metagenomes were dealt with consistently, and the low abundance of nonmicrobial reads was low. Forward and reverse reads were then combined into a single fasta file for each sample using the fq2fa command from IDBA-UD (80).

Ten profiles of each microbiome were described. These included four taxonomic levels (species, genus, family, and phylum), four functional profiles (Superfocus 1, Superfocus 2, Superfocus 3, and Carbohydrate functions, which are a subset of HUMAnN2 output), the bacteriocin gene profile, and the antimicrobial resistance gene profile.

Kaiju v1.5.0 (81) was used to assign taxonomy to the reads, using the NCBI BLAST nonredundant protein database, including fungi and microbial eukaryotes, discarding taxa with a relative abundance of <0.1%. This setting was chosen since other studies have shown a high false-positive discovery rate below this threshold (82). All percentages reported at all taxonomic levels are percentages of the assigned reads only. Species-level assignment was updated for lactic acid bacteria, as previously described (83). Superfocus (84) was used to assign functionality to the reads. Superfocus assigns reads to homologues gene families to determine functionality. It collapses these gene families to higher levels of organization for a more generic function. Superfocus level 1 is the highest level of organization, followed by levels 2 and 3, with 3 having the most specific function. Data Set S1, sheet 1, shows the complete list of microbes and their relative abundance for each food. The phylogenetic tree of L. lactis was created in GraPhlAn (85), using the StrainPhlAn (86) output, which used Metaphlan2 (87) taxonomic assignment.

Statistical analyses were carried out in R-3.2.2 (88) using vegan (89). Analysis of similarities (ANOSIM) was carried out between each metadata category containing six or more samples (Data Set S1, sheet 6). The Benjamini-Hochberg false discovery rate was applied to the ANOSIM results. The linear discriminant analysis (LDA) effect size (LEfSe) (90) method was used to determine whether any taxa or pathways were differentially abundant between groups (see Text S1 in the supplemental material).

10.1128/mSystems.00522-20.1 TEXT S1 LDA of differences between different food substrates. Download Text S1, DOCX file, 0.01 MB. Copyright © 2020 Leech et al. 2020 Leech et al. This content is distributed under the terms of the Creative Commons Attribution 4.0 International license.

### Antimicrobial resistance.

Antimicrobial resistome analysis was performed by aligning paired-end metagenomes reads against the MEGAres database (v1.0.1) (91). To reduce type I errors, this database was first manually curated to remove any genes corresponding to antimicrobial resistance arising from point mutations. The alignment was performed using the –very-sensitive-local preset of Bowtie2 (v2.3.4). The Resistome Analyser tool (https://github.com/cdeanj/resistomeanalyzer) was used to format the output, and the results were normalized for sequencing depth across samples as counts per million reads (CPM).

### Bacteriocin assignment.

Bacteriocin assignment was performed with the BLAST analysis of the bacteriocin genome mining tool (BAGEL) of the predicted genes with the Prodigal tool against the BAGEL4 bacteriocin databases (92).

### Carbohydrate pathways.

The carbohydrate function was assigned to reads with the HUMAnN2 pipeline (93), which assigned the function based on the ChocoPhlan databases and genes based on UniRef (94). To further simplify the exploration of the abundance data of the gene family were grouped into the functional category Gene Ontology (GO), specifically carbohydrate-related functions, performing a more in-depth analysis.

### Metagenomic assembled genomes.

Metagenome assembly was carried out using IDBA-UD. MetaBAT 2 (95) was used for genome binning, with default settings. CheckM (96) was implemented to check the quality of metagenome assembled genomes (MAGs). Low-quality MAGs, i.e., <80% completeness and/or >10% contamination, were removed from downstream analysis. Kaiju (81) and PhyloPhlAn (97) were used to assign taxonomy to the MAGs. The average nucleotide identity (ANI) of MAGs to reference genomes, which were downloaded from RefSeq (98), was calculated using FastANI (64). Putatively novel MAGs were assigned as potentially new species using the ANI threshold described previously (78). The phenotypes of MAGs were predicted using Traitar (63). MAGs were annotated using Prokka (99).

### PLS-DA analyses.

Partial least-squares discriminant analysis (PLS-DA) plots were generated using the KODAMA R package (v1.5) (100). Default parameters of the KODAMA software were used on species from the taxonomic profile with the semisupervised constraining of data ordination according to the fermentation process of samples. The final visualization of data was performed in R (v3.5.1) using ggplot2 (v3.1.1) (101).

### PHAGC screening.

Shotgun sequences for 16 nonfermented dairy samples were downloaded from ENA (study accession number PRJEB31110) with a median of 18,041 reads per sample, after removing Bos taurus reads. The 16 dairy samples were as follows: raw tanker milk, *n* = 2; skimmed milk powder, *n* = 6; pasteurized skimmed milk, *n* = 4; and raw silo whole milk, *n* = 4. The fermented- and nonfermented-food sequences were then assigned UniRef clusters (90) using the HUMAnN2 software (93). Using the UniRef clusters obtained from HUMAnN2 output, the presence or absence of clusters shown to influence the potential health-promoting properties of bacteria was determined (13, 60, 102). The list of search terms can be found in Data Set S1, sheet 7. The total numbers of PHAGCs present in each food were binned into one of the following three categories: survival, modulation, and colonization. A heatmap was created using Pheatmap (103). The rows of the heatmap were scaled, so that the values are comparative between the foods and not an absolute count of the numbers of gene clusters found in each food.

### Data availability.

All raw reads can be accessed from the ENA under project accession number PRJEB35321.
