# Supplementary figures and images for: Fermented-Food Metagenomics Reveals Substrate-Associated Differences in Taxonomy and Health-Associated and Antibiotic Resistance Determinants
Source: mSystems. 2020 Nov 10;5(6):e00522-20. doi: 10.1128/mSystems.00522-20 (PMC7657593; doi:10.1128/mSystems.00522-20)

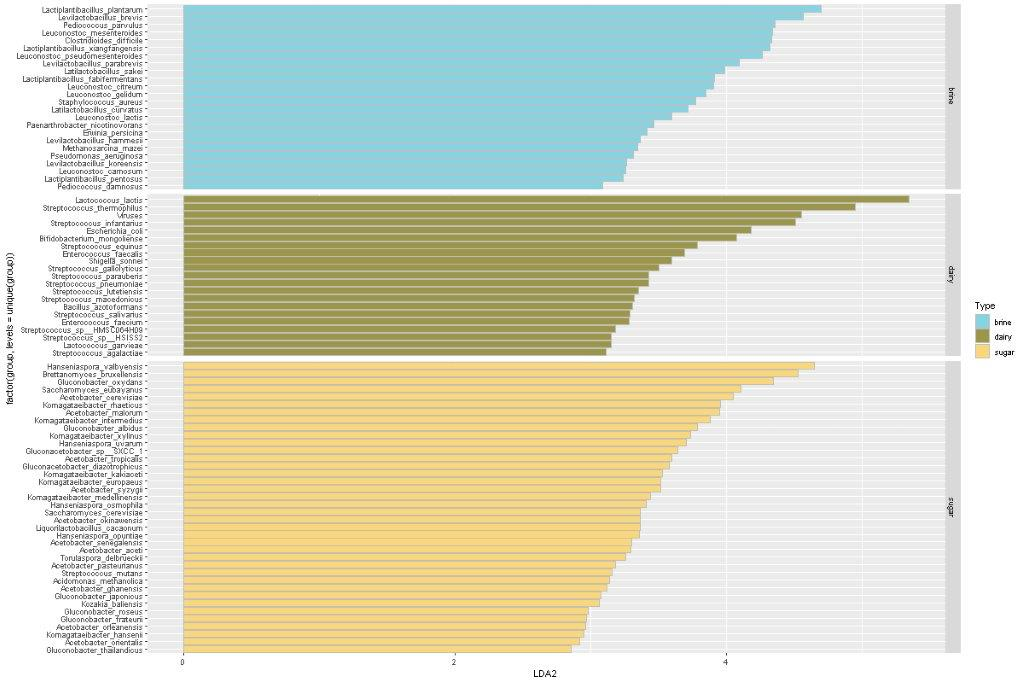

Supplement: FIG S2 [file mSystems.00522-20-sf002.tif]
