# Supplementary material for: Fermented-Food Metagenomics Reveals Substrate-Associated Differences in Taxonomy and Health-Associated and Antibiotic Resistance Determinants
Source: mSystems. 2020 Nov 10;5(6):e00522-20. doi: 10.1128/mSystems.00522-20 (PMC7657593; doi:10.1128/mSystems.00522-20)
Supplement: TEXT S1 [file mSystems.00522-20-s0001.docx]

# Supplementary Material

### Food types defined by Family level taxonomy

Linear discriminant analysis (LDA) effect size (LEfSe) method ([54](#_ENREF_54)) revealed 351 taxonomic and functional characteristics that were differentially abundant across the food types. This included 82 species, 34 genera, 27 families and 8 phyla (**Supplementary Figure 2**). This included observations that *Lactococcus lactis* (LDA = 5.64), *Streptococcus thermophilus* (LDA = 5.24) and viruses (LDA = 4.86) were more abundant in dairy samples, *Lactiplantibacillus plantarum* (LDA = 4.99) and *L. brevis* (LDA = 4.84) were more abundant in brine foods and *Hanseniospora valbyensis* (LDA = 4.94), *Brettanomyces bruxellensis* (LDA = 4.83) and *Gluconobacter oxydans* (LDA = 4.64) were more abundant in sugar samples. At the family level these corresponded to higher abundances of *Lactobacillaceae* (LDA = 5.68) in brine foods, *Streptococcaceae* (LDA = 5.92) in dairy foods and *Acetobacteraceae* (LDA = 5.5) in sugar foods.

From a functional perspective, 200 pathways at the three SuperFocus levels were different across the three food types. These differences include phage related functions (LDA = 4.78, SF1) and adhesion (LDA = 3.59, SF2) enriched pathways in dairy, osmotic stress (LDA = 4, SF2) in brine samples, and vitamins (LDA = 4.89, SF1), fatty acids (LDA = 2.75, SF2) and multidrug resistance efflux pumps in sugar foods (LDA = 1.06, SF3).
